# Propolis Ethanolic Extract Attenuates D-gal-induced C2C12 Cell Injury by Modulating Nrf2/HO-1 and p38/p53 Signaling Pathways

**DOI:** 10.3390/ijms24076408

**Published:** 2023-03-29

**Authors:** Songhao Tian, Huiting Zhao, Hongru Guo, Wei Feng, Conglin Jiang, Yusuo Jiang

**Affiliations:** 1 College of Animal Science, Shanxi Agricultural University, Taigu 030801, China; 2 Department of Medical Laboratory, Fenyang College of Shanxi Medical University, Fenyang 032200, China; 3 College of Life Sciences, Shanxi Agricultural University, Taigu 030801, China

**Keywords:** PEE, muscle atrophy, aging, D-gal, C2C12

## Abstract

Previous study has shown that propolis ethanolic extract (PEE) has a protective effect on aging skeletal muscle atrophy. However, the exact molecular mechanism remains unclear. This study aimed to investigate the effect of PEE on D-galactose (D-gal)-induced damage in mouse C2C12 cells. The results revealed that PEE increased the viability of senescent C2C12 cells, decreased the number of senescence-associated β-galactosidase (SA-β-Gal)-positive cells and promoted the differentiation of C2C12 cells. PEE resisted oxidative stress caused by D-gal by activating the Nrf2/HO-1 signaling pathway and maintained the differentiation ability of C2C12 cells. PEE inhibited apoptosis by suppressing p38 phosphorylation and reducing p53 expression. In summary, our findings reveal the molecular mechanism by which PEE protects D-gal-induced C2C12 cells, providing a theoretical basis for the development of PEE for the alleviation of muscle atrophy.

## 1. Introduction

Skeletal muscle constitutes 30–40% of body mass and plays a critical role in body functions such as movement, thermoregulation and metabolism [1,2]. Aging is one of the factors that contributes to skeletal muscle atrophy. Skeletal muscle atrophy in the elderly severely impairs health, reduces quality of life and leads to increased mortality [3]. The presence of advanced glycation end-products (AGEs) accumulated in skeletal muscle tissue has been demonstrated in the elderly and manifests itself as a cause of skeletal muscle dysfunction [4]. AGEs cause muscle atrophy through AGE receptor-mediated downregulation of the Akt signaling pathway by AMPK [5]. AGEs damage the protein and fat components of muscle tissue by promoting the production of damaging molecules such as free radicals and inflammatory cytokines [6]. D-galactose (D-gal) is a reducing sugar that reacts readily with amino acid free amines in proteins and peptides in vitro and in vivo to form AGEs [7]. D-gal induces oxidative stress, and the accumulation of ROS under oxidative stress conditions leads to lipid peroxidation and glucose oxidation reactions, which also produce AGEs [8,9]. Increased AGEs hasten the aging process. D-gal mouse and rat models have been widely used in aging studies and drug testing [10,11]. D-gal-induced mouse and cellular senescence models have also been used for the study of muscle atrophy due to aging [12,13,14,15]. C2C12 cells have been used to evaluate the potential of various natural and pharmaceutical compounds in alleviating insulin resistance, anti-aging, muscle atrophy and hyperlipidemia [16]. MyoD and Myogenin (MyoG) expression stimulates differentiation of myogenic cells into myotubes during myogenesis [17]. At the end stage of muscle differentiation, late differentiation markers such as myosin heavy chain (MHC) are induced [18]. When the C2C12 cell line was cultured in myogenic differentiation medium, the cells rapidly differentiated into myotubes [19].

Propolis is a sticky plant substance collected by bees and may include different types of plant secretions [20]. As a functional food, propolis is used to improve health and prevent disease. Propolis ethanolic extracts (PEEs) obtained by grinding, dissolving in ethanol and filtering the original propolis retain most of the active ingredients in propolis. In in vivo and in vitro studies, propolis has demonstrated antioxidant, anti-inflammatory, hepatoprotective and immunomodulatory effects [21]. Poplar type propolis exhibits very good antioxidant activity and anti-AGE activity, and will be a promising anti-aging drug candidate [22]. Flavonoid fractions in propolis effectively block the synthesis of AGEs by capturing dicarbonyl intermediates [23]. Previous studies have found that propolis has the ability to alleviate muscle atrophy, but its molecular mechanisms remain elusive [6,14]. The Nrf2/Keap1 signaling pathway plays a crucial role in preventing the onset of cancer in normal cells by shielding them from oxidative damage [24]. Its antioxidant properties make this pathway pivotal in the development of chemoresistance in various cancer cells [25,26,27]. Coffee phenethyl ester (CAPE) in propolis activates the Nrf2 pathway, thereby balancing the redox state through the Nrf2/HO-1 pathway. Flavonoids in propolis regulate intracellular ROS levels, antioxidant enzyme activity, nuclear factor erythroid 2-related factor 2 (Nrf2) nuclear translocation and hemeoxygenase 1 (HO-1) expression, which help maintain the balance of antioxidant and anti-inflammatory effects in cells [28]. Several studies have shown that p38MAPK can be activated by intracellularly generated ROS and that activation of p38MAPK affects the regulation of downstream transcription factors, such as p53 and Nrf2, to control the expression of downstream pro- and anti-apoptotic genes in response to many extracellular stimuli, including oxidative stress [29]. Muscle atrophy is closely associated with high levels of AGEs produced by oxidative stress. p38MAPK-mediated P53 and Nrf2 pathways are involved in oxidative stress-induced apoptosis. Therefore, we hypothesized that PEE may protect against D-gal-induced C2C12 cell injury by regulating the ROS-p38-p53/Nrf2 signaling pathway. The aim of this study was to investigate the molecular mechanism of PEE protection against D-gal-induced oxidative damage in C2C12 cells in mice and to provide a basis for evaluating the role of PEE in alleviating muscle atrophy.

## 2. Results

### 2.1. Effect of PEE on D-Gal-Induced C2C12 Cell Viability and Senescence

**Figure 1:**
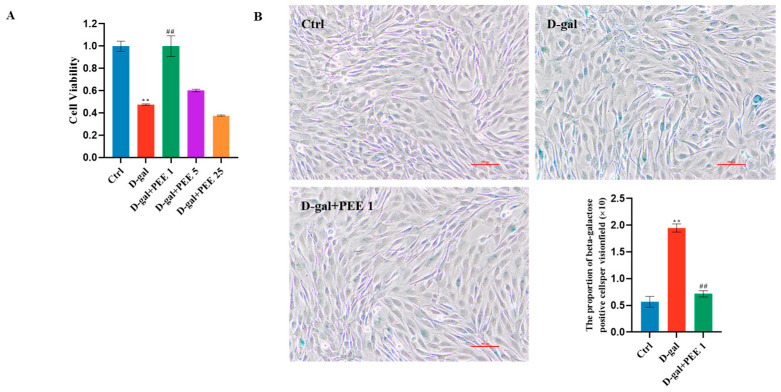
Propolis ethanolic extract (PEE) increased the viability of D-galactose (D-gal)-treated C2C12 cells and attenuated the senescence of C2C12 cells. (**A**) Effect of PEE (1, 5, 25 µg/mL) on the viability of D-gal-treated C2C12 cells. (**B**) Senescence-associated β-galactosidase (SA-β-Gal) staining and quantification for C2C12 cells (scale bar = 100 μm). All data are expressed as mean ± SEM (n = 3). ** *p* < 0.01 vs. Ctrl; ^##^ *p* < 0.01 vs. D-gal.

In this study D-gal was used to induce a muscle atrophy model in C2C12 cells. The cytotoxicity of D-gal on C2C12 was first detected by CCK-8, and 111 mM D-gal treatment for 48 h was selected as the experimental condition for D-gal to evaluate the protective effect of PEE on D-gal-treated C2C12 cells (Figure S1A,B). To determine the effect of PEE on D-gal-induced C2C12 cells, D-gal-treated C2C12 cells were incubated with 0, 1, 5 and 25 µg/mL PEE for 48 h. Cell survival was significantly higher (*p* < 0.01) after 1 µg/mL PEE treatment compared to the D-gal-treated group (Figure 1A). Therefore, 1 µg/mL PEE was chosen as the experimental concentration of PEE. Then, we investigated the effect of PEE on the senescence of D-gal-treated C2C12 cells using senescence-associated β-galactosidase (SA-β-Gal) staining. The number of SA-β-Gal positive cells was significantly lower (*p* < 0.01) after PEE treatment compared to the D-gal-treated group (Figure 1B), indicating that PEE significantly improved the senescence of D-gal-treated C2C12 cells.

### 2.2. Effect of PEE on Differentiation Ability of D-Gal-Treated C2C12 Cells

**Figure 2:**
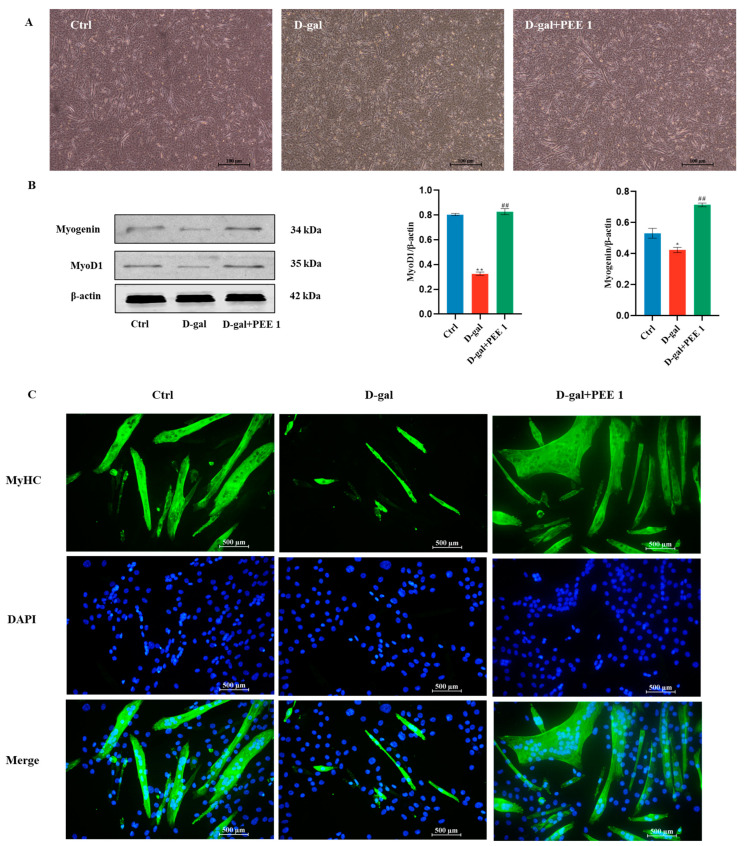
PEE alleviated the impairment of differentiation capacity of D-gal-treated C2C12 cells. (**A**) Morphological changes in the number and size of myotubes after PEE treatment of C2C12 cells (scale bar = 100 μm). (**B**) Western blot and quantification for MyoD, and Myogenin. (**C**) The fluorescence staining for myosin heavy chain (MHC), indicator of Myotubular fusion (scale bar = 500 μm). All data are expressed as mean ± SEM (n = 3). * *p* < 0.05 and ** *p* < 0.01 vs. Ctrl; ^##^ *p* < 0.01 vs. D-gal.

D-gal treatment inhibits C2C12 cell differentiation, and we next determined whether PEE plays an important role in the differentiation of C2C12 cells treated with D-gal. PEE treatment increased the number and size of fused myotubes (Figure 2A,C). Protein blotting results showed that D-gal down-regulated the expression of myogenic markers MyoD and Myogenin (*p* < 0.05), but PEE treatment significantly up-regulated these markers (*p* < 0.01) (Figure 2B). These results suggest that PEE can attenuate the impaired differentiation ability of C2C12 cells caused by D-gal.

### 2.3. PEE Reduces Oxidative Stress in D-Gal-Treated C2C12 Cells

**Figure 3:**
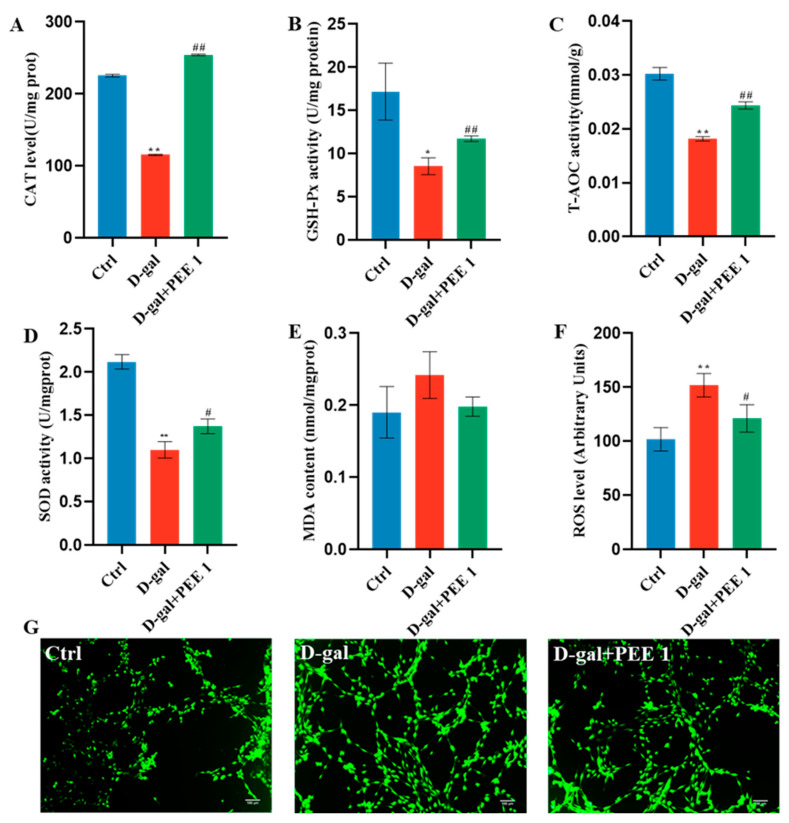
PEE attenuated ROS production and improved redox homeostasis in D-gal-treated C2C12 cells. Levels of CAT (**A**), GSH-Px (**B**), T-AOC activity (**C**), SOD (**D**), MDA (**E**) and ROS (**F**) in C2C12 cells. (**G**) Representative images of intracellular ROS in C2C12 cells stained with DCFH-DA after PEE treatment (scale bar = 100 μm). All data are expressed as mean ± SEM (n = 3). * *p* < 0.05 and ** *p* < 0.01 vs. Ctrl; ^#^ *p* < 0.05 and ^##^ *p* < 0.01 vs. D-gal.

To evaluate whether PEE supplementation could reduce oxidative stress caused by D-gal, antioxidant enzyme activity and ROS levels in C2C12 were analyzed. D-gal treatment significantly reduced the levels of CAT, GSH-PX, T-AOC and SOD in C2C12 cells compared to the control (*p* < 0.05) (Figure 3). In addition, using the DCFH-DA probe, we also found that D-gal treatment significantly increased ROS levels in cells (*p* < 0.01) (Figure 3F,G). Compared with the D-gal-treated group, PEE treatment significantly increased the levels of CAT, GSH-PX, T-AOC and SOD in C2C12 cells (*p* < 0.05) (Figure 3), and significantly decreased the levels of ROS in C2C12 cells (*p* < 0.05) (Figure 3F,G). D-gal and PEE treatment did not affect the level of MDA in C2C12 cells. In conclusion, these results suggest that PEE has a protective effect against D-gal-induced oxidative stress but does not regulate MDA levels in C2C12 cells.

### 2.4. PEE Attenuates D-Gal-Induced C2C12 Cell Injury by Activating the Nrf2/HO-1 Pathway

**Figure 4:**
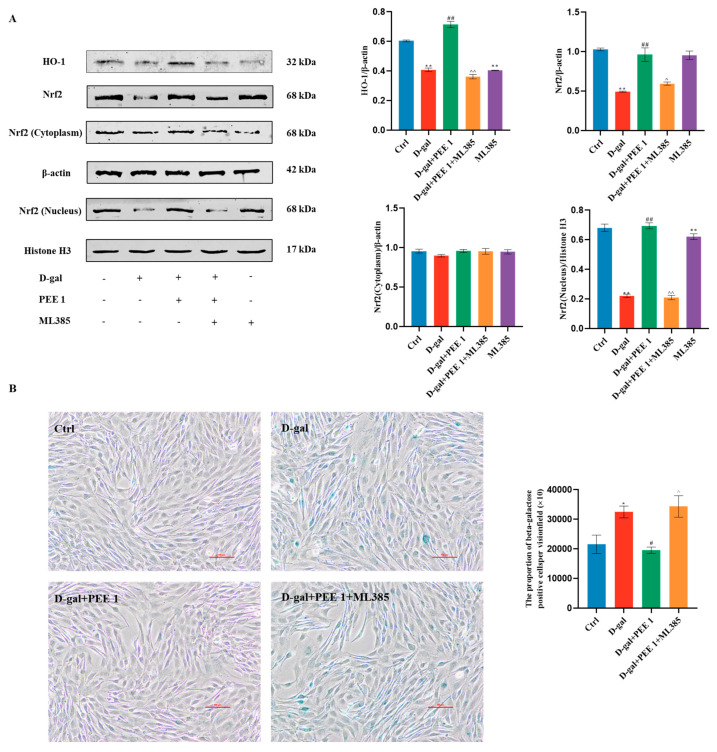

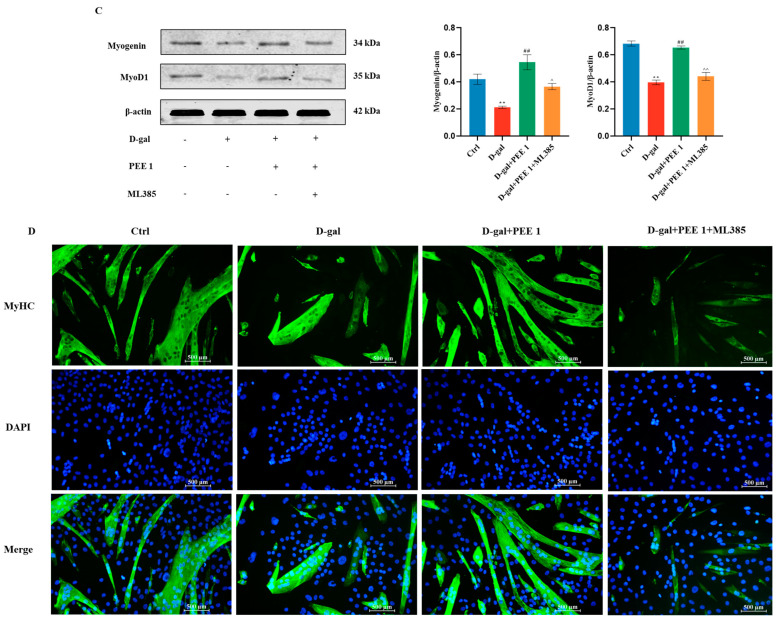
PEE protected C2C12 from oxidative damage through Nrf2/HO−1 signaling pathway. (**A**) ML385 treatment reversed the role of PEE in promoting nuclear translocation of Nrf2 in C2C12 cells. (**B**) ML385 treatment reversed the alleviating effect of PEE on senescence in C2C12 cells. (**C**) ML385 treatment abolished the ameliorative effect of PEE on D-gal-induced differentiation capacity of C2C12 cells. (**D**) ML385 treatment reversed the myotubular fusion-promoting effect of PEE. All data are expressed as mean ± SEM (n = 3). * *p* < 0.05 and ** *p* < 0.01 vs. Ctrl; ^#^ *p* < 0.05 and ^##^ *p* < 0.01 vs. D-gal; ^^^ *p* < 0.05 and ^^^^ *p* < 0.01 vs. D-gal + PEE 1.

Nrf2 is an important transcription factor in the cellular antioxidant system, and *HO-1* is a target gene downstream of Nrf2. PEE significantly upregulated the protein levels of Nrf2 and HO-1 in C2C12 cells compared with the D-gal-treated group (*p* < 0.01) (Figure 4A). In addition, we also found that PEE significantly up-regulated Nrf2 levels in the nucleus of C2C12 cells (*p* < 0.01) (Figure 4A), while it had no effect on Nrf2 expression in the cytoplasm. These results suggest that PEE may counteract D-gal-induced oxidative stress in C2C12 by promoting nuclear translocation of Nrf2. To further investigate whether PEE regulates D-gal-induced oxidative damage in C2C12 cells mediated through the Nrf2/HO-1 pathway, it was verified with ML385, an Nrf2-specific inhibitor. Western blotting results showed that the addition of ML385 reversed the effects of PEE in promoting nuclear translocation of Nrf2 and upregulating HO-1 protein levels (Figure 4A). It was also found that addition of ML385 abolished the improvement of PEE on D-gal-induced C2C12 cell senescence (Figure 4B). The pro-differentiation effect of PEE on C2C12 cells was reversed after the addition of ML385. (Figure 4C,D). In conclusion, these results provide valid evidence that PEE protects D-gal-induced C2C12 cells from oxidative damage through activation of the Nrf2/HO-1 signaling pathway.

### 2.5. PEE Inhibits D-Gal-Induced Apoptosis in C2C12 Cells through p38/p53 Pathway

**Figure 5:**
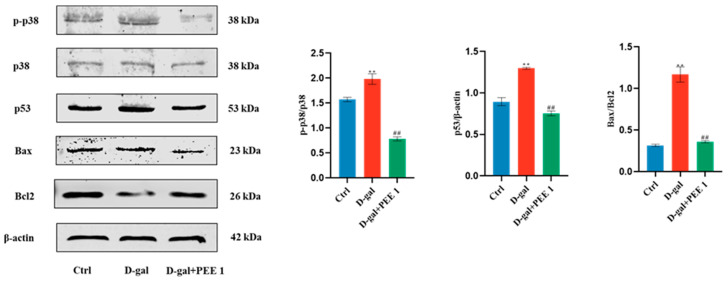
PEE inhibited D-gal-induced apoptosis in C2C12 cells by the p38/p53 signaling pathway. All data are expressed as mean ± SEM (n = 3). ** *p* < 0.01 vs. Ctrl; ^##^ *p* < 0.01 vs. D-gal.

p38/p53 signaling pathways are closely related to apoptosis. To investigate whether PEE regulates D-gal-induced apoptosis in C2C12 cells through these pathways, we examined the protein expression of the p38/p53 signaling pathway. Compared with the control group, D-gal treatment significantly increased the phosphorylation level of p38 while significantly increasing the expression of p53 (*p* < 0.01) (Figure 5). p53 activation is capable of leading to apoptosis, and our results show that D-gal treatment significantly increased the Bax/Bcl2 ratio (*p* < 0.01) (Figure 5). The D-gal-induced p38 phosphorylation, p53 expression and elevated Bax/Bcl2 ratio were reversed by the addition of PEE. These results suggest that PEE may reduce D-gal-induced apoptosis in C2C12 cells by inhibiting the p38/p53 signaling pathway.

## 3. Discussion

We used C2C12 cells treated with a concentration of 111 mM D-gal for 48 h to establish a model of senescent muscle cells, and these results are consistent with the findings of Chen et al. [30] and Yang et al. [12], indicating that muscle atrophy can be modeled in vitro with D-gal. SA-β-Gal is a widely used biomarker associated with the senescence phenotype and is capable of detecting cells that have undergone aging. Senescent myogenic cells show G0/G1 phase cell cycle arrest, DNA damage and elevated SA-β-Gal activity [31]. D-gal treatment resulted in an increase in the number of SA-β-Gal positive cells, indicating that D-gal successfully induced a senescent cell model. PEE treatment increased cell survival and decreased the number of SA-β-Gal positive cells, indicating that PEE has a better anti-senescence effect and can protect the cells from the damage caused by D-gal.

The differentiation capacity of senescent myogenic cells is impaired [31]. D-gal causes impaired differentiation capacity of C2C12 cells by downregulating the expression of mitochondrial calcium uptake family member 3 (MICU3), a member of the mitochondrial calcium uptake family, in C2C12 cells [12]. Our study also found that D-gal caused a decrease in the differentiation capacity of C2C12 cells, which is consistent with the results of the previous study. It was found that bone marrow mesenchymal stem cell (MSC) supernatants elevated MyoG and MyoD protein levels and had a promotional effect on C2C12 cell differentiation [32]. In the present study, PEE was found to promote the expression of MyoG and MyoD, thereby restoring the differentiation ability of D-gal-injured C2C12 cells, suggesting that PEE has a better pro-differentiation function, offering the possibility that PEE can be used to prevent muscle atrophy in the elderly.

A large body of evidence suggests that oxidative stress plays an important role in the signaling pathways that regulate protein synthesis and protein degradation in skeletal muscle, and that oxidative stress caused by disease and prolonged muscle inactivity leads to skeletal muscle atrophy [33]. SOD, CAT and GSH-Px play important roles in the process of oxidative stress and can help the body to scavenge excess ROS and protect cells from oxidative stress. MDA is produced in the presence of excess ROS that cause lipid peroxidation in the cell membrane and, to some extent, can also be used to reflect the extent of oxidative damage in the cell. PEE was able to alleviate oxidative damage in senescent C2C12 cells (Figure 3), suggesting that the antioxidant effect of PEE may be able to play a role in alleviating muscle atrophy.

Nrf2 is a ubiquitous major transcription factor that is usually present in the cytoplasm as an inactive complex and binds to the blocking molecule Keap1. Under oxidative stress, Nrf2 dissociates from Keap1 and translocates to the nucleus, which in turn induces the expression of antioxidant genes and provides protection to the organism [34]. In our study, we found that in D-galactose-induced C2C12 cells, PEE promoted nuclear translocation of Nrf2 and facilitated the expression of the downstream antioxidant gene *HO-1*. The pro-differentiation and anti-aging effects of PEE were reversed upon addition of the Nrf2-specific inhibitor ML385. Nrf2/Keap1 pathway activation may have an anti-apoptotic effect [35,36]. Nrf2 inactivation is necessary for apoptosis in the context of extensive cellular damage induced by oxidative stress [37]. Narasimhan et al. found that Nrf2-deficient mice had reduced muscle differentiation and significant activation of skeletal muscle apoptotic pathways [38]. Nrf2 inhibitor ML385 reverses the effect of MSCs to reduce the number of SA-β-Gal positive cells in D-gal-induced senescent CD4^+^ T cells [39]. Our experimental results confirm that PEE may protect against D-galactose-induced C2C12 cell injury via the Nrf2/HO-1 pathway by a mechanism that enhances antioxidant enzyme activity and maintains cell differentiation capacity in an Nrf2-dependent manner.

**Figure 6:**
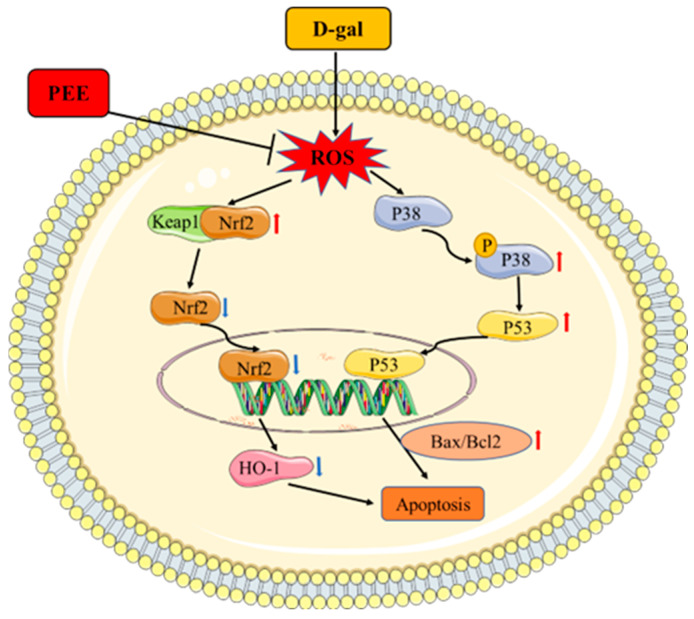
The protective mechanism of PEE on D-gal induced C2C12 cell injury.

PEE alleviates muscle atrophy in mice by reducing isoproterenol levels in muscle and inhibiting muscle cell apoptosis [14]. Isoproterenol induces activation of p38 MAPK [40]. In our study, we found that PEE inhibits the phosphorylation of p38 and suppresses the expression of downstream p53 and pro-apoptotic genes. p38 is a major member of the MAPK signaling pathway, which is activated by phosphorylation and plays a role in mediating apoptosis [41]. Previous studies have shown that activation of p38 MAPK affects the expression of the downstream transcription factor p53 to control the expression of downstream pro-apoptotic genes [42]. Our results suggest that PEE can inhibit p38 activation and regulate D-gal-induced apoptosis in C2C12 cells by modulating the p53 pathway. Taken together, our results suggest that PEE may block D-gal-induced apoptosis in C2C12 cells by activating the Nrf2/HO-1 pathway, increasing antioxidant enzyme activity and inhibiting P38/P53 pathway activation together (Figure 6). These results provide a rationale for the use of PEE to alleviate muscle atrophy in the elderly.

## 4. Materials and Methods

### 4.1. Materials and Reagents

High-sugar DMEM medium (PYG0073), 0.25% trypsin solution (AR1007) and CCK-8 kit (AR1199) were purchased from Boster Biological Technology Co., Ltd. (Wuhan, China). Fetal bovine serum (FBS) (SA201.01) was obtained from CellMax Technology (Beijing) Co. (Beijing, China). Penicillin mixture (100×) (P1400) was purchased from Beijing Solarbio Science & Technology Co., Ltd. (Beijing, China). The PEE was generously donated by Prof. Li-Ming Wu of the Institute of Apicultural Research, Chinese Academy of Agricultural Sciences, and was analyzed via high performance liquid chromatography-diode array detection/quadrupole time-of-flight mass spectrometry (HPLC-DAD/Q-TOF-MS) to determine the PEE components (Table S1) [43]. The D-gal (478518-63-7) was obtained from Sigma-Aldrich (St Louis, MI, USA). SA-β-Gal staining kit (C0602) was acquired from Beyotime Biotechnology (Shanghai, China). Malondialdehyde (MDA) (A003-3-1), peroxidase (CAT) (A007-1-1), glutathione peroxidase (GSH-Px) (A005-1-2), superoxide dismutase (SOD) (A001-1-2), total antioxidant capacity (T-AOC) (A015-2-1), reactive oxygen species (ROS) (E004-1-1) biochemical kits were provided by Nanjing Jiancheng Bioengineering Institute (Nanjing, China). The Nrf2-specific inhibitor ML385 (HY-100523) was purchased from MedChemExpress LLC (Shanghai, China). Information on the antibodies used in the experiment is shown in Table 1.

### 4.2. Cell Culture

Mouse C2C12 myogenic cell line was obtained from American Typical Culture Collection (Manassas, VA, USA) and grown in an incubator at 37 °C and 5% CO_2_. The growth medium (GM) was DEME containing 10% (*v*/*v*) fetal bovine serum and 1% (*v*/*v*) penicillin/streptomycin. D-gal was dissolved in PBS and diluted to the appropriate concentration at the time of use. Referring to Wang et al. [44], the ethanolic extract of propolis was redissolved in ethanol, filtered through a 0.22 μm filter membrane and stored in a 4 °C refrigerator and diluted to the corresponding concentration when used. To test the effect of D-gal on C2C12 cells, cells were incubated with different doses of D-gal (55.5, 111, 166.5, 222 or 277.5 mM). To test the effect of PEE on D-gal-induced senescent cells, different doses of PEE (1, 5, 25 μg/mL) were co-incubated with the cells for 12 h and then replaced with medium containing D-gal to continue the culture. The Nrf2-specific inhibitor ML385 (5 μM) [11] was added simultaneously with PEE to observe the effect of the inhibitor on the cells. To test the effect of PEE on C2C12 differentiation, the growth medium was changed to a differentiation medium (DM) (DMEM + 2% (*v*/*v*) horse serum + 1% (*v*/*v*) penicillin/streptomycin) when the cells grew to 80% of the culture flask to induce cell differentiation.

### 4.3. CCK-8 Assay for Cell Viability

Log phase C2C12 cells were inoculated uniformly in 96-well plates, and the cells were grouped according to the experimental needs. After adding the drug treatment according to the experimental requirements, CCK-8 solution was added and incubated in 5% CO_2_ and 37 °C incubator for 15 min, after which the OD value was measured at 450 nm. Cell survival rate = [(OD of experimental wells—OD of blank wells)/(OD of control wells—OD of blank wells)] × 100%.

### 4.4. SA-β-Gal Staining

SA-β-Gal staining solution fixative was added to the cell medium and the cells were fixed at room temperature for 15 min. Cells were then washed 3 times with PBS for 3 min each. Then 1mLSA-β-Gal staining working solution was added to each well and incubated overnight at 37 °C without CO_2_ until some of the cells turned blue. Cells were observed under the microscope and photographed, and positive cells were counted.

### 4.5. Determination of Antioxidant Activity in C2C12 Cells

The cells were broken by ultrasonic treatment. The levels of MDA, SOD, GSH-Px, T-AOC and CAT in C2C12 cells were determined according to the kit procedure. The BCA kit was used to determine the protein content in the samples at 562 nm.

### 4.6. Measurement of ROS Content

Cells cultured in 6-well plates were added to 1 mL of DCFH-DA probe at a concentration of 10 M and incubated at 37 °C for 60 min. After washing twice with PBS, the 6-well plates were photographed under a fluorescence microscope. ImageJ software (1.51 k Version, NIH, Bethesda, MD, USA) was used to analyze the green fluorescence intensity in the field of view.

### 4.7. Western Blotting

The cell samples were homogenized completely with a homogenizer after adding protein lysis solution (RIPA), centrifuged at 12,000 rpm for 10 min at 4 °C via freezing centrifuge and the supernatant was taken in a metal bath at 100 °C for 10 min to obtain total cellular proteins. Cytoplasmic and nuclear protein extraction was performed according to the kit instructions. After protein concentration was determined via BCA kit, proteins were separated via SDS-PAGE electrophoresis and then transferred to PVDF membrane. Then, the membrane was blocked with 0.05% skim milk powder at room temperature for 1 h. Primary antibodies were added and incubated overnight at 4 °C on a shaker, washed three times with TBST, and secondary antibodies were incubated for 1 h under protection from light, and protein gray values were evaluated with the Odyssey scanning system. The ratio of the target protein to β-actin or Histone H3 was used to calculate the relative expression of the protein.

### 4.8. Statistical Analysis

Data are expressed as mean ± SEM. Comparisons between different groups were performed via Student’s t-test using SPSS 20.0 (SPSS Inc., Chicago, IL, USA). The difference at *p* < 0.05 level was considered significant, and the difference at *p* < 0.01 level was remarkably significant.

## 5. Conclusions

As a functional food, propolis has the potential to alleviate muscle atrophy. In this study, we investigated the protective effect of PEE on D-gal-induced C2C12 cell injury and its mechanism using D-gal-induced C2C12 cells as a model. PEE alleviates D-gal-induced C2C12 injury through Nrf2/HO-1 and p38/p53 pathways. PEE maintains normal muscle cell function through antioxidant and anti-apoptotic effects. Our study provides a new perspective on using PEE to prevent aging-induced muscle atrophy.

## Figures and Tables

**Table 1:** Antibodies information.

Antibodies	Cat No.	Company	Source	Dilution Ratio
β-actin	l102	Bioworld Technology, Inc, Nanjing, China	Rabbit	1:20,000
HO-1	A1346	ABclonal Technology Co., Ltd., Wuhan, China	Rabbit	1:1000
Nrf-2	A1820	ABclonal Technology Co., Ltd., Wuhan, China	Rabbit	1:1000
Histone H3	A2348	ABclonal Technology Co., Ltd., Wuhan, China	Rabbit	1:1000
Myogenin	A17427	ABclonal Technology Co., Ltd., Wuhan, China	Rabbit	1:1000
Bcl-2	WL01556	Wanleibio, Shenyang, China	Rabbit	1:500
Bax	WL01637	Wanleibio, Shenyang, China	Rabbit	1:500
p38	WL00764	Wanleibio, Shenyang, China	Rabbit	1:500
p-p38	WLP1576	Wanleibio, Shenyang, China	Rabbit	1:500
p53	WL01919	Wanleibio, Shenyang, China	Rabbit	1:500
MyoD1	WL04662	Wanleibio, Shenyang, China	Rabbit	1:500
MHC	MF20	Developmental Studies Hybridoma Bank, Iowa City, IA, USA	Mouse	1:20
IgG H&L/FITC	bs-0296G-FITC	Bioss Antibodies, Beijing, China	Goat	1:100
IRDye^®^800CW	C926-32211	LI-COR Biosciences, Lincoln, NE, USA	Goat	1:20,000

## Data Availability

The data presented in this study are available upon request from the corresponding author.

## Supplementary Materials

The following supporting information can be downloaded at: https://www.mdpi.com/article/10.3390/ijms24076408/s1.
