# Supplementary material for: Propolis Ethanolic Extract Attenuates D-gal-induced C2C12 Cell Injury by Modulating Nrf2/HO-1 and p38/p53 Signaling Pathways
Source: Int J Mol Sci. 2023 Mar 29;24(7):6408. doi: 10.3390/ijms24076408 (PMC10094417; doi:10.3390/ijms24076408)
Supplement: Supplementary file 1 [file ijms-24-06408-s001.zip › Supplementary materials-update.pdf]

Table S1 Composition of Chinese Propolis (CP) Ethanol Extract[1]

Compound number	Compound name	RT（min）	Content in CP（mg/g raw propolis）
1	Protocatechuic acid	30.98	29.48
2	Luteolin	26.33	4.03
3	Apigenin	31.42	3.87
4	Chrysin	30.70	3.57
5	3,4-Dimethoxycinnamic acid	21.05	3.15
6	trans-Isoferulic acid	16.79	3.10
7	(Pinocembrin)	29.87	2.69
8	3-O-acetylpinobanksin	30.19	2.11
9	Caffeic acid	7.41	2.10
10	p-Coumaric acid	12.15	1.85
11	Pinobanksin	25.64	1.71
12	Ferulic acid	14.65	1.65
13	Galangin	27.76	1.56
14	Gallic acid	22.88	0.67
15	Kaempferol	27.40	0.63
16	Cinnamic acid	22.93	0.56
17	Quercetin	25.54	0.30
18	Protocatechuic acid	3.05	0.25
19	Hesperitin	26.40	0.06
20	Vanillic acid	6.24	0.02

1. K. Wang, X. Jin, Q. Li, A. Sawaya, R. K. Le Leu, M. A. Conlon, L. Wu and F. Hu, Propolis from Different Geographic Origins Decreases Intestinal Inflammation and Bacteroides spp. Populations in a Model of DSS-Induced Colitis, *Mol. Nutr. Food Res.*, 2018, **62**, e1800080.

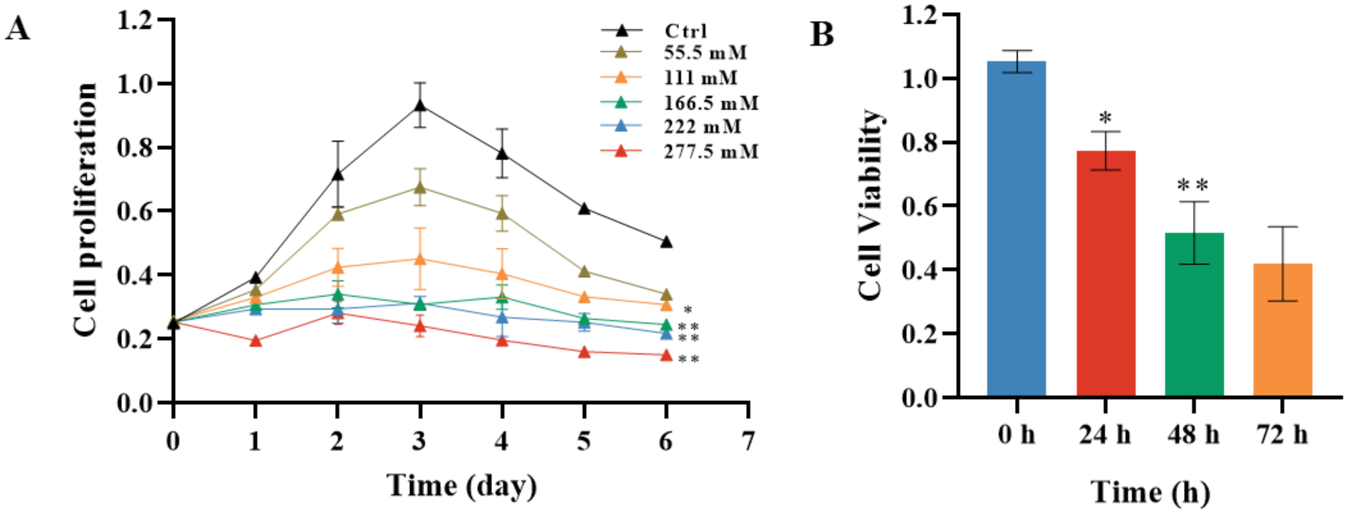

Fig. S1 Establishment of a D-gal-induced senescent cell model. (A) Effect of D-gal (55.5-277.5 mM) on the proliferation of C2C12 cells. (B) Effect of D-gal (111 mM) treatment on C2C12 cell viability at different time periods. All data are expressed as mean ± SEM (n = 3). * $P < 0.05$ and ** $P < 0.01$.
